# Associations Between Microbiota Awareness, Healthy Eating Attitude, and Sociodemographic Factors in University Students

**DOI:** 10.1002/fsn3.70280

**Published:** 2025-05-13

**Authors:** Emine Kocyigit, Ayçıl Özturan Şirin, Nilüfer Ozkan

**Affiliations:** ^1^ Department of Nutrition and Dietetics Faculty of Health Sciences, Ordu University Ordu Türkiye; ^2^ Department of Nutrition and Dietetics Faculty of Health Sciences, Aydın Menderes University Aydın Türkiye

**Keywords:** awareness, healthy eating attitude, microbiota, prebiotics, probiotics

## Abstract

The global epidemic of diet‐related chronic diseases emphasizes the significance of the human gut microbiota in dietary physiological effects and the etiology of chronic diseases. This research aimed to assess the relationship between microbiota awareness, healthy eating attitudes, and sociodemographic factors in university students. Two hundred forty‐two university students participated in this descriptive and analytical study in Aydın and Ordu, Türkiye. University students completed a general information form, anthropometric measures, the Attitude Scale for Healthy Nutrition, the Microbiota Awareness Scale, and a probiotic and prebiotic food frequency questionnaire. In total, 48 (19.8%) male and 194 (80.2%) female students participated in the study. Nutrition and dietetics students had higher total scores and sub‐factor scores in ASHN and microbiota awareness and lower body mass index compared to other departments (*p* < 0.05). A positive and significant correlation was found between the Attitude Scale for Healthy Nutrition and Microbiota Awareness Scale total scores. Conversely, a negative statistically significant correlation was observed between body mass index and the total score of the Microbiota Awareness Scale (*p* < 0.05). The frequency of probiotic and prebiotic food consumption was higher in nutrition and dietetics students compared to other departments. A higher Attitude Scale for Healthy Nutrition, studying in the nutrition and dietetic department, knowing the term microbiota, and self‐assessment on microbiota knowledge as good were associated with increased awareness of microbiota (*p* < 0.05). Given this positive association, integrating healthy nutrition, microbiota, probiotics, and prebiotics into the educational curricula for all university departments, scientific conferences, and academic research may enhance microbiota awareness among university students.

## Graphical abstract

Nutrition and dietetics students had higher total scores and sub‐factor scores in ASHN and microbiota awareness and lower body mass index compared to other departments. A positive and significant correlation was found between ASHN and Microbiota Awareness Scale total scores. Conversely, a negative statistically significant correlation was observed between BMI and the total score of the Microbiota Awareness Scale. A higher ASHN, studying in the nutrition and dietetic department, knowing the term microbiota, and self‐assessment on microbiota knowledge as good were associated with increased awareness of microbiota.

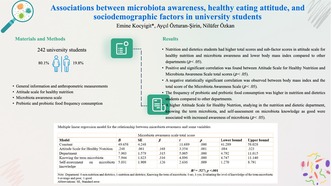

## 1 Introduction

The microorganisms living in the human body referred to as “microbiota”, and their interactions with the eukaryotic host have emerged as an important topic of research in recent years (Ghanbari et al. 2024; Liang et al. 2023; Zmora et al. 2019). The term microbiota in the Human Microbiome Project describes all populations of microorganisms (bacteria, fungi, archaea, viruses, and protozoa) that colonize specific areas within the host (Turnbaugh et al. 2007). Immediately after birth, this community begins developing shape and quickly grows into an active biodiversity. In the first 2–3 years of life, bifidobacteria serve as a crucial element in stabilizing the diversity of the microbiome. As time passes, the microbial composition in adult humans becomes more diverse and complex. The human gut predominantly consists of the *Bacteroidota* and *Bacillota* bacterial phyla, accounting for approximately 90% of the total bacterial abundance in the majority of individuals, while a small number of individuals exhibit dominance by *Pseudomonadota* (Ling et al. 2022; Manor et al. 2020; Martin et al. 2023). The gut microbiome consists of the entire genome of all bacteria residing in the gut, significantly influencing the body's nutritional, metabolic, physiological, and immunological functions (Garcia et al. 2023).

Significant heterogeneity in microbiome composition exists across individuals. Numerous endogenous and exogenous variables affect the composition of the microbiota, including geographical origin, genetics, method of delivery at birth, age, lifestyle, diet, antibiotic treatment, and prior illnesses (Davarci and Davarci 2025). Diet is an important indicator of microbiota, acting as a both an immediate and long‐term regulator of gut microbiota. The diet supplies essential nutrients to the gut microbiota, which affects the absorption, metabolism, and storage of these nutrients. Additionally, the gut microbiota generates new substances that provide feedback to the host, potentially influencing various physiological processes significantly (Rinninella et al. 2019; Schlechte et al. 2023). The Mediterranean diet and intermittent fasting positively affect metabolism by influencing gut microbiota composition and function. However, high intake of alcohol, processed foods, and food additives has been linked to the onset of dysbiosis, as well as inflammatory and metabolic disorders (Ozkul et al. 2020; Turpin et al. 2022; Zhang et al. 2025).

Prebiotics and probiotics are the most popular therapeutic agents for maintaining a healthy microbiome (Piccioni et al. 2023). The International Scientific Association for Probiotics and Prebiotics (ISAPP), the Food and Agriculture Organization, and the World Health Organization describe probiotics as “live microorganisms that, when provided in sufficient quantities, confer health advantages to the host” (Hill et al. 2014). Fermented milk products are the primary dietary source of probiotics. Yogurt, sour cream, kefir, various kinds of cheese, boza, soy products (miso, natto, tempeh), unheated fermented vegetables, cereals, meat products (salami, pepperoni, sausages), some beers, and kombucha are probiotic fermented foods. ISAPP describes prebiotics as “a substrate specifically utilized by host bacteria that confer health advantages.” Cereals include wheat, barley, and oats; legumes such as dry beans, chickpeas, lentils, and kidney beans; vegetables including onions, garlic, chicory, asparagus, spinach, leeks and Jerusalem artichokes; as well as bananas and forest fruits are abundant in pulp with prebiotic properties (Cosme et al. 2022; Gibson et al. 2017; Marco et al. 2021).

The growing body of microbiota research has elucidated the relationship between microbiota and health (Çıtar Dazıroğlu et al. 2024; Ertaş Öztürk et al. 2024). The significance of awareness about microbiota health and the impact of probiotic and prebiotic foods on this health is growing. This study aimed to evaluate the microbiota awareness levels of university students who started a new period in their lives and whose eating habits were also affected by the effects of many changing conditions, and to examine the factors affecting microbiota awareness levels.

## 2 Materials and Methods

### 2.1 Study Design and Participants

This descriptive and analytical study was conducted with 242 university students (80.2% female, 19.2% male, 18–30, mean age 21.8 ± 3.70 years) between March and June 2024. The study comprised university students from the Faculty of Health Sciences, Faculty of Tourism, and Faculty of Education at Ordu University, along with those from the Faculty of Health Sciences at Aydın Adnan Menderes University. Power analysis was performed to determine the size of the study sample. It was calculated that the study should be conducted with 252 students at a statistically significant level (*d*: 0.08, *p* < 0.05 at 90% confidence interval). Ten university students with incomplete information were excluded from the study. The inclusion criteria specified that university students not have chronic or psychological diseases, have a history of severe childhood trauma, not be pregnant or breastfeeding, and be willing to participate in the study. Ethical permission was obtained from the Ordu University Social Sciences and Humanities Research Ethics Committee (Meeting Number: 10; Decision Number: 2023‐245; Date: 28.11.2023). The study was carried out in compliance with the ethical principles outlined in the Declaration of Helsinki. Written consent was obtained from the students who volunteered to participate in the study.

### 2.2 Collection of Data

University students who volunteered for the study received a questionnaire through face‐to‐face interviews. Several measurements were used to assess the relationship between microbiota awareness, attitudes towards healthy eating, and the influencing factors, including age, gender, department of study, number of main and snack meals, and body mass index (BMI). The questionnaire included a general information form, anthropometric measurements, the Microbiota Awareness Scale, the Attitude Scale for Healthy Nutrition (ASHN), and prebiotic and probiotic food frequency consumption.

### 2.3 General Information and Anthropometric Measurements

The general information form included questions evaluating sociodemographic characteristics (age [year], gender [male/female], grade of education), number of main meals and snacks, hearing and knowing the term microbiota, prebiotic, and probiotic, self‐assessment on microbiota, prebiotic, and probiotic knowledge. The university students self‐reported their height (cm) and body weight (kg). The BMI was calculated by dividing body weight by the square of height and assessed according to the World Health Organization's BMI classification. The classification of BMS is as follows: a value below 18.50 kg/m^2^ indicates underweight, a range from 18.50 to 24.99 kg/m^2^ indicates normal weight, a value between 25.0 and 29.99 kg/m^2^ is considered overweight, and a measurement above 30.0 kg/m^2^ is categorized as obese (WHO 2003).

### 2.4 Attitude Scale for Healthy Nutrition

Tekkurşun Demir and Cicioğlu developed the Attitude Scale for Healthy Nutrition (ASHN), and the same researchers conducted its validity and reliability analyses (Demir and Cicioğlu 2019). The ASHN consists of 21 questions and four sub‐factors. The Cronbach's alpha internal consistency coefficient of the scale is 0.90. On a five‐point Likert scale, the questions are answered (“1” Strongly Disagree, “2” Disagree, “3” Undecided, “4” Agree, and “5” Strongly Agree). Items 6, 7, 8, 9, 10, 11, 17, 18, 19, 20, and 21 are reverse‐coded. The scale's factors were defined in this manner: the first factor was information on nutrition, the second factor was emotion for nutrition, the third factor was positive nutrition, and the fourth factor was malnutrition. The lowest score that can be obtained from the scale is 21, and the highest score is 105. Participants scoring 21 points were classified as very low, those scoring between 23 and 42 points as low, scores from 43 to 63 points as moderate, 64 to 84 points as high, and scores ranging from 85 to 105 points showed an ideally high attitude towards healthy eating. A higher score on the scale reflects an elevated attitude towards healthy eating among individuals.

### 2.5 Microbiota Awareness Scale

The Microbiota Awareness Scale, created by Külcü and Önal in 2022, aims to assess the microbiota awareness levels among adults, with subsequent validation and reliability testing conducted (Külcü and Önal 2022). The reliability of the scale, as indicated by the calculated Cronbach Alpha coefficient, was 0.852. The scale comprises four sub‐dimensions (General Information, Product Information, Chronic Disease, Probiotic, and Prebiotic) and 20 questions. The first 16 questions are based on a five‐point Likert scale (1 = strongly disagree, 2 = disagree, 3 = decided, 4 = agree, 5 = strongly agree). Questions 17 and 18 of the scale consist of five‐choice information questions, with each correct response awarded 1 point and each wrong response also receiving 1 point. The 19th and 20th questions of the scale are open‐ended and evaluated as follows: no correct answers receive 1 point, one correct answer receives 2 points, two correct answers receive 3 points, three correct answers receive 4 points, and four or more correct answers receive 5 points. High scores on the scale, without an established cut‐off point, indicate an elevated level of microbiota awareness.

### 2.6 Prebiotic and Probiotic Food Frequency Consumption

The Probiotic and Prebiotic Food Consumption Frequency form, which the researchers created by reviewing the current literature. We assessed the frequency of dietary intake of prebiotic and probiotic foods using a 34‐item Food Frequency Questionnaire, which inquired about the frequency of food and beverage consumption over the last 6 months. The questionnaire includes meat and meat products (red meat, poultry, fermented sucuk, dried meat, etc.), dairy and fermented products (milk, soy milk, tofu, yogurt, etc.), cereals and oil seeds (oatmeal, granola bar, whole grains, nuts, etc.), vegetables and fruits (banana, pineapple, plums, onion, etc.), and beverages (red wine, coffee, vegetable juice, etc.). The frequency in the FFQ was evaluated as none, 2–3 times a month or less, 3–4 times a week, 2–3 times a day, and once a day.

### 2.7 Statistical Analysis

The statistical analysis was conducted using the Statistical Package for the Social Sciences (version 26.0) (IBM Corp., Armonk, NY, USA). The normal distribution of the data was assessed through visual methods (histogram and probability graphs) and analytical techniques (Shapiro–Wilk test). Descriptive statistics were presented as frequency and percentage for categorical variables and mean and standard deviation for numerical variables. An independent *t*‐test was used for the normal distribution of data. Mann–Whitney *U* and Kruskal Wallis tests were used to distribute non‐normal data. Bonferroni correction was applied for multiple pairwise comparisons. Chi‐square tests were used for categorical data. Relationships between numerical variables were given with the Spearman correlation coefficient. Multiple linear regression using an enter method was used to study the factors associated with microbiota awareness. Microbiota awareness scale total score was used as the dependent variable, department (code: 0‐non nutrition and dietetics, 1‐nutrition and dietetics), knowing the term microbiota (code: 0‐no, 1‐yes), self‐assessment on microbiota knowledge (code: 0‐average and poor, 1‐good), and ASHN total score were used as independent variables in the model. The results were statistically evaluated at a *p* < 0.05 significance level.

## 3 Results

The general characteristics of the participants in the study are given in Table 1. The mean age of the individuals was 21.8 ± 3.70 (80.2% female, 19.2% male). 50.0% of the students studied in the Nutrition and Dietetic department, 21.5% in Elementary Mathematics Teaching, 14.9% in Cookery, and 13.6% in Gastronomy and Culinary Arts. Among the students, 5.8% were in the first grade, 21.1% were in the second, 40.8% were in the third, and 33.1% were in the fourth. The mean BMI of the students was 24.0 ± 14.70 kg/m^2^. According to BMI classification, most individuals (61.3%) were within the normal range. 46.3% of students knew the term microbiota, 76.9% the term prebiotics, and 85.5% the term probiotics. Furthermore, 55.4% of the students evaluated their knowledge of microbiota, 23.1% of prebiotics, and 18.2% of probiotics as poor. The mean score of the students in the ASHN was determined as 79. ± 9.87. The Microbiota Awareness Scale total score and the scores of General information, Product information, Chronic disease, and Probiotic and prebiotic subscales were determined as 72.8 ± 12.55, 24.7 ± 4.02, 10.5 ± 3.45, 18.6 ± 3.64, and 19.0 ± 3.7, respectively.

**TABLE 1:** General characteristics of the participants (*n*: 242).

	*n*	%
Gender		
Male	48	19.8
Female	194	80.2
Department		
Nutrition and dietetics	121	50.0
Non nutrition and dietetics	121	50.0
Grade of education		
1st	14	5.7
2nd	51	21.1
3rd	97	40.1
4th	80	33.1
Receiving nutrition education		
Yes	210	86.8
No	32	13.2
Hearing the term microbiota		
Yes	130	53.7
No	112	46.3
Hearing the term prebiotic		
Yes	221	91.3
No	21	8.7
Hearing the term probiotic		
Yes	227	93.8
No	15	6.2
Knowing the term microbiota		
Yes	112	46.3
No	130	53.7
Knowing the term prebiotic		
Yes	186	76.9
No	56	23.1
Knowing the term probiotic		
Yes	207	85.5
No	35	14.5
Self‐assessment on microbiota knowledge		
Good	27	11.2
Average	81	33.5
Poor	134	55.4
Self‐assessment on prebiotic knowledge		
Good	57	23.6
Average	129	53.3
Poor	56	23.1
Self‐assessment on probiotic knowledge		
Good	62	25.6
Average	136	56.2
Poor	44	18.2
BMI classification		
Underweight	28	11.6
Normal	152	63.1
Overweight	40	16.6
Obese	21	8.7

	$\bar{x}$ ± SD	
Age (years)	21.8	3.70
Number of main meals	2.3	0.52
Number of snacks	1.5	0.83
BMI (kg/m^2^)	24.0	14.70
Attitude Scale for Healthy Nutrition		
Total score	73.3	10.36
Information on nutrition	21.7	3.00
Emotion for nutrition	17.0	4.10
Positive nutrition	17.0	3.84
Malnutrition	17.7	3.79
Microbiota Awareness Scale		
Total score	72.8	12.55
General information	24.7	4.02
Product information	10.5	3.45
Chronic disease	18.6	3.64
Probiotic and prebiotic	19.0	3.70

*Note:* Non nutrition and dietetics: Elementary mathematics teaching (21.5%), Cookery (14.9%), Gastronomy and culinary arts (13.6%).

Abbreviations: BMI, body mass index; HNAS, Healthy Nutrition Attitude Scale.

Table 2 shows the evaluation of variables according to the department. The prevalence of hearing and knowing the terms microbiota, prebiotic, and probiotic was significantly higher among students in the nutrition and dietetics department (*p* < 0.001). A significantly higher number of students in the nutrition and dietetics department evaluated their knowledge of microbiota, prebiotics, and probiotics as good (*p* < 0.001). The BMI levels were notably elevated among university students not enrolled in nutrition and dietetics programs (*p* < 0.001), with a higher number of overweight and obese individuals observed in this group. The nutrition and dietetics students had considerably higher ASHN overall scores and Microbiota Awareness Scale total and subscale scores (*p* < 0.001, Tables 3 and 4).

**TABLE 2:** Evaluation of variables according to the department.

	Deparment		*p*
	Nutrition and dietetics	Non nutrition and dietetics	
Receiving nutrition education			
Yes	114 (94.2)	96 (79.3)	0.001^a^
No	7 (5.8)	25 (20.7)	
Hearing the term microbiota			
Yes	107 (88.4)	23 (19.0)	< 0.001^a^
No	14 (11.6)	98 (81.0)	
Hearing the term prebiotic			
Yes	119 (98.3)	102 (84.3)	< 0.001^a^
No	2 (1.7)	19 (15.7)	
Hearing the term probiotic			
Yes	119 (98.3)	108 (89.3)	0.003^a^
No	2 (1.7)	13 (10.7)	
Knowing the term of microbiota			
Yes	98 (81.0)	14 (11.6)	< 0.001^a^
No	23 (19.0)	107 (88.4)	
Knowing the term of prebiotic			
Yes	116 (95.9)	70 (57.9)	< 0.001^a^
No	5 (4.1)	51 (42.1)	
Knowing the term of probiotic			
Yes	118 (97.5)	89 (73.6)	< 0.001^a^
No	3 (2.5)	32 (26.4)	
Self‐assessment on microbiota knowledge			
Good	24 (19.8)	3 (2.5)	< 0.001^a^
Average	64 (52.9)	17 (14.0)	
Poor	33 (27.3)	101 (83.5)	
Self‐assessment on prebiotic knowledge			
Good	48 (39.7)	9 (7.4)	< 0.001^a^
Average	66 (54.5)	63 (52.1)	
Poor	7 (5.8)	49 (40.5)	
Self‐assessment on probiotic knowledge			
Good	52 (43.0)	10 (8.3)	< 0.001^a^
Average	64 (52.9)	72 (59.5)	
Poor	5 (4.1)	39 (32.2)	
Number of main meals	2.3 ± 0.47	2.3 ± 0.57	0.725
Number of snacks	1.6 ± 0.83	1.4 ± 0.82	0.032^c^
BMI (kg/m^2^)	22.0 ± 4.02	26.0 ± 20.24	< 0.001^b^
BMI classification			
Underweight	21 (17.4)	7 (5.8)	0.002^a^
Normal	80 (66.1)	72 (60.0)	
Overweight	14 (11.6)	26 (21.7)	
Obese	6 (4.9)	15 (12.5)	
Attitude Scale for Healthy Nutrition			
Total score	77.3 ± 8.69	69.3 ± 10.40	< 0.001^b^
Information on nutrition	22.7 ± 2.68	20.6 ± 2.94	< 0.001^c^
Emotion for nutrition	18.3 ± 3.84	15.8 ± 4.00	< 0.001^c^
Positive nutrition	17.8 ± 3.69	16.2 ± 3.82	0.001^c^
Malnutrition	18.5 ± 3.06	16.9 ± 4.26	0.002^c^
Microbiota Awareness Scale			
Total score	81.0 ± 10.11	65.0 ± 9.30	< 0.001^c^
General information	26.6 ± 3.29	22.7 ± 3.71	< 0.001^c^
Product information	12.6 ± 2.97	8.4 ± 2.52	< 0.001^c^
Chronic disease	20.5 ± 3.37	16.7 ± 2.86	< 0.001^c^
Probiotic and prebiotic	21.1 ± 2.98	17.0 ± 3.23	< 0.001^c^

Abbreviation: BMI, body mass index.

^a^ Chi‐square test.

^b^ Independent *t* test.

^c^ Mann–Whitney *U* test.

**TABLE 3:** Evaluation of microbiota awareness of university students according to some variables.

	General information	*p*	Product information	*p*	Chronic disease	*p*	Probiotic and prebiotic	*p*	Total score	*p*
	$\bar{x}$ ± SD		$\bar{x}$ ± SD		$\bar{x}$ ± SD		$\bar{x}$ ± SD		$\bar{x}$ ± SD	
Gender										
Male	23.1 ± 25.03	0.002^a^	9.5 ± 3.11	0.017^a^	17.4 ± 3.23	0.009^a^	17.8 ± 3.75	0.008^a^	67.8 ± 11.89	0.003^a^
Female	25.0 ± 3.92		10.8 ± 3.49		18.9 ± 3.68		19.3 ± 3.64		74.0 ± 12.43	
Department										
Nutrition and dietetics	26.6 ± 3.29	< 0.001^a^	12.6 ± 2.97	< 0.001^a^	20.5 ± 3.37	< 0.001^a^	21.1 ± 2.98	< 0.001^a^	80.7 ± 10.11	< 0.001^a^
Non nutrition and dietetics	22.7 ± 3.71		8.4 ± 2.52		16.7 ± 2.86		17.0 ± 3.23		64.8 ± 9.30	
Receiving nutrition education										
Yes	25.0 ± 4.05	< 0.001^a^	10.7 ± 3.47	< 0.001^a^	20.3 ± 3.54	< 0.001^a^	20.7 ± 3.44	< 0.001^a^	79.8 ± 11.21	< 0.001^a^
No	22.6 ± 3.13		9.1 ± 2.97		16.6 ± 2.66		17.1 ± 3.01		64.7 ± 8.57	
Knowing the term microbiota										
Yes	26.8 ± 3.22	< 0.001^a^	12.7 ± 3.06	< 0.001^a^	20.7 ± 3.43	< 0.001^a^	21.2 ± 3.02	< 0.001^a^	81.5 ± 10.13	< 0.001^a^
No	22.8 ± 3.69		8.6 ± 2.48		16.8 ± 2.74		17.1 ± 3.15		65.3 ± 9.12	
Knowing the term prebiotic										
Yes	25.6 ± 3.56	< 0.001^a^	12.7 ± 3.06	< 0.001^a^	20.7 ± 3.43	< 0.001^a^	21.2 ± 3.02	< 0.001^a^	65.3 ± 9.12	< 0.001^a^
No	21.7 ± 4.05		8.6 ± 2.48		16.8 ± 2.74		17.1 ± 3.15		76.1 ± 11.35	
Knowing the term probiotic										
Yes	25.3 ± 3.61	< 0.001^a^	11.0 ± 3.42	< 0.001^a^	19.0 ± 3.55	< 0.001^a^	15.6 ± 3.35	< 0.001^a^	75.0 ± 11.60	< 0.001^a^
No	21.0 ± 4.35		7.7 ± 1.98		16.0 ± 3.13		19.7 ± 3.33		59.6 ± 9.55	
Self‐assessment on microbiota knowledge										
Good	27.6 ± 3.32^x^	< 0.001	13.7 ± 3.19^x^	< 0.001	21.8 ± 3.62^x^	< 0.001	22.48 ± 3.47^x^	< 0.001	85.6 ± 11.22^x^	< 0.001
Average	26.4 ± 3.21^x^		12.3 ± 3.01^x^		20.3 ± 3.14^x^		20.8 ± 2.77^y^		79.8 ± 9.85^y^	
Poor	23.0 ± 3.21^y^		8.8 ± 2.68^y^		16.9 ± 2.97^y^		17.25 ± 3.23^z^		66.0 ± 9.58^z^	
Self‐assessment on prebiotic knowledge										
Good	26.7 ± 3.21^x^	< 0.001	12.5 ± 3.25^x^	< 0.001	20.5 ± 3.94^x^	< 0.001	21.8 ± 3.24^x^	< 0.001	81.6 ± 11.43^x^	< 0.001
Average	25.0 ± 3.61^y^		10.6 ± 3.37^y^		18.7 ± 3.21^y^		19.2 ± 3.05^y^		73.5 ± 10.77^y^	
Poor	21.8 ± 4.08^z^		8.2 ± 2.25^z^		16.5 ± 3.13^z^		15.9 ± 3.16^z^		62.2 ± 9.65^z^	
Self‐assessment on probiotic knowledge										
Good	26.6 ± 3.36^x^	< 0.001	12.7 ± 3.28^x^	< 0.001	20.5 ± 4.04^x^	< 0.001	21.9 ± 3.22^x^	< 0.001	81.6 ± 11.69^x^	< 0.001
Average	24.8 ± 3.49^y^		10.3 ± 3.24^y^		18.5 ± 3.09^y^		18.9 ± 2.95^y^		72.5 ± 10.28^y^	
Poor	21.3 ± 4.38^z^		8.1 ± 2.41^z^		16.2 ± 3.26^z^		15.5 ± 3.32^z^		61.2 ± 10.39^z^	
BMI classification										
Underweight	25.7 ± 3.28	0.224	10.6 ± 3.10	0.085	19.9 ± 3.10	0.099	19.4 ± 3.63	0.116	75.5 ± 11.00	0.117
Normal	24.7 ± 4.23		10.8 ± 3.44		18.6 ± 3.94		19.3 ± 3.86		73.4 ± 13.19	
Overweight	24.0 ± 3.81		9.4 ± 3.43		18.3 ± 3.06		18.2 ± 3.48		69.9 ± 11.27	
Obese	23.9 ± 3.69		9.9 ± 3.46		17.8 ± 2.88		18.3 ± 2.96		69.9 ± 11.41	
ASHN total score										
Moderate	22.0 ± 4.45^x^	< 0.001	8.7 ± 2.31^x^	< 0.001	16.7 ± 3.06^x^	< 0.001	16.7 ± 3.41^x^	< 0.001	64.0 ± 10.64^x^	< 0.001
High	24.9 ± 3.78^y^		10.5 ± 3.31^y^		18.7 ± 3.59^y^		19.1 ± 3.63^y^		73.2 ± 12.08^y^	
Ideally high	26.5 ± 3.21^y^		13.3 ± 3.90^z^		20.4 ± 3.69^y^		21.5 ± 2.69^z^		81.7 ± 10.66^z^	

*Note:* There is a significant difference between x, y, z groups.

Abbreviations: ASHN, Attitude Scale for Healthy Nutrition; BMI, body mass index.

^a^ Mann–Whitney *U* test, other tests Kruskal Wallis test.

**TABLE 4:** Evaluation of the relationship between microbiota awareness and some variables (correlation coefficient = *r*).

	Microbiota awareness scale total score		
	Nutrition and dietetics	Non nutrition and dietetics	Overall
Age (years)	0.209*	0.042	0.025
BMI (kg/m^2^)	0.045	0.087	−0.156*
Attitude Scale for Healthy Nutrition total score	0.370**	0.175	0.469**
Sub‐factors			
Information on nutrition	0.425**	0.303*	0.537**
Emotion for nutrition	0.081	−0.028	0.212**
Positive nutrition	0.315**	0.169	0.338**
Malnutrition	0.210*	0.070	0.250**

*Note:* Spearman correlation coefficient.

Abbreviation: BMI, body mass index.

* *p* < 0.05.

** *p* < 0.001.

A statistically significant difference was found concerning sex, department, receipt of nutrition education, knowing about microbiota, prebiotics, and probiotics, as well as knowledge levels related to microbiota, prebiotics, and probiotics about the overall score of the “Microbiota Awareness Scale,” including sub‐factor scores for general information, product information, chronic disease, and probiotic and prebiotic (*p* < 0.05). Individuals with good knowledge of the microbiota have higher scores in chronic disease, probiotics, prebiotics, and the overall “Microbiota Awareness Scale” compared to those with moderate and poor knowledge (*p* < 0.001). The general information, product information, chronic illness, probiotic and prebiotic scores, and the total score of the “Microbiota Awareness Scale” in those with proficient knowledge are higher than those of individuals with moderate and poor knowledge (*p* < 0.001). No statistically significant differences were observed in the total score of the Microbiota Awareness Scale and its sub‐factor scores when analyzed according to BMI classification (*p* > 0.05). Students with moderate healthy eating attitudes have lower scores on general information, product information, probiotic and prebiotic, and the total score of the “Microbiota Awareness Scale” than those who have high and ideally high healthy eating attitudes (*p* < 0.001; statistics obtained as a result of Bonferroni correction are not shown in the table). Significant positive correlations were found between the total score of ASHN, information on nutrition, emotion for nutrition, and positive nutrition and the total score of the microbiota awareness scale (*p* < 0.001). In contrast, negative correlations were found for the malnutrition sub‐factor of ASHN and the total score of the microbiota awareness scale (*p* < 0.001). Negative correlations were observed for the BMI value of students and the microbiota awareness scale total score (*p* < 0.05).

**FIGURE 1:**
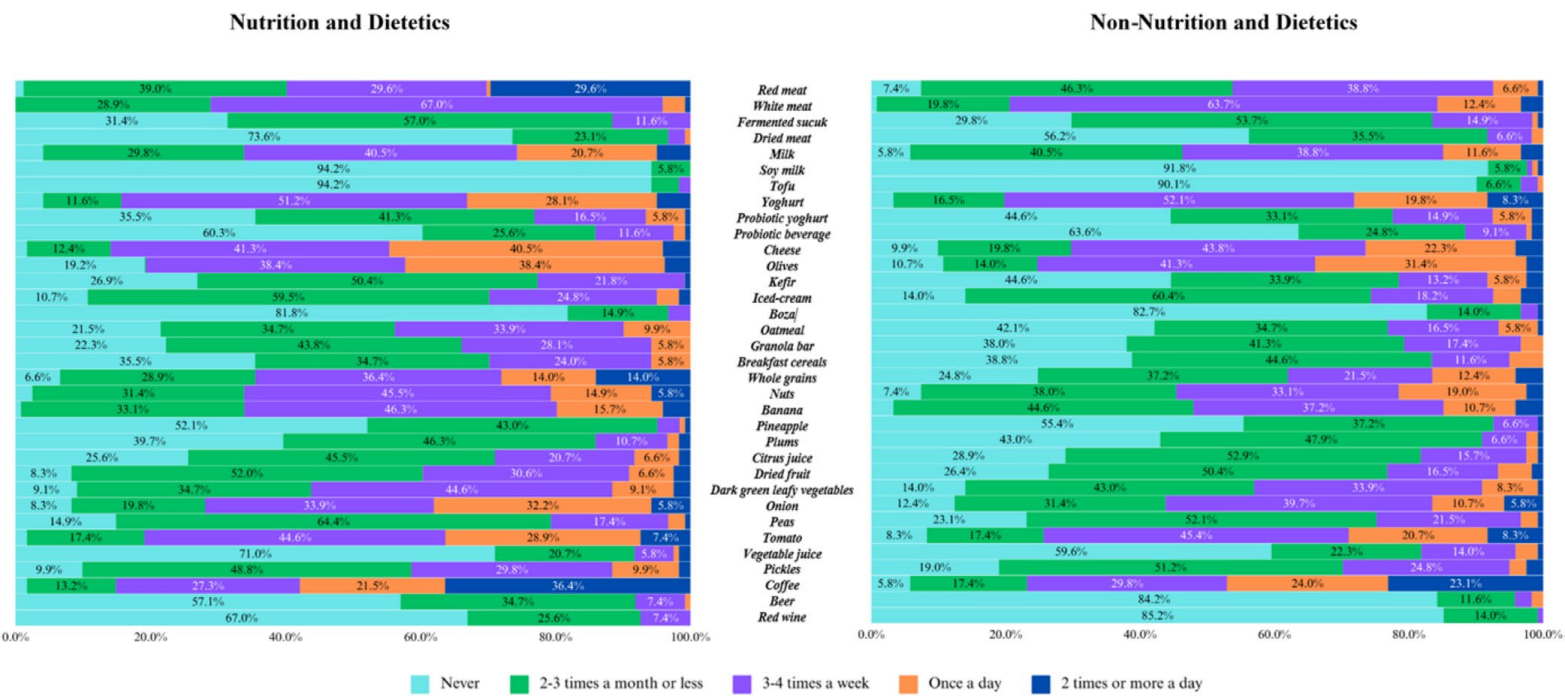
Prebiotic and probiotic food frequency consumption among university students.

Figure 1 demonstrates the frequency of consumption of prebiotic and probiotic foods among university students. The consumption frequency of red meat, white meat, and dried meat among nutrition and dietetics students is notably higher than that of others. Cheese, kefir, oats, granola, dried fruit, onions, pickles, beer, and red wine were found to be consumed more frequently by students who did not study nutrition and dietetics (*p* < 0.05; statistics obtained as a result of Chi‐square test are not shown in the figure).

When the factors (ASHN total score, department, knowing the term of microbiota, self‐assessment on microbiota knowledge) related to microbiota awareness total scores were evaluated with multiple linear regression analysis, the model was significant (*R* ^2^: 0.527, *p* < 0.001). Microbiota awareness was related to the ASHN total score (*p* < 0.05), nutrition and dietetics department (*p* < 0.001), knowing the term of microbiota (*p* < 0.001), and self‐assessment on microbiota knowledge as good (*p* < 0.001) (Table 5).

**TABLE 5:** Multiple linear regression model for the relationship between microbiota awareness and some variables.

Model	*B*	Microbiota awareness scale total score					
		SE	*β*	*t*	*p*	Lower bound	Upper bound
Constant	49.658	4.248		11.689	0.000	41.289	58.028
Attitude Scale for Healthy Nutrition	0.248	0.061	0.168	3.354	0.001	0.084	0.323
Department	7.903	1.579	0.315	5.005	0.000	4.792	11.015
Knowing the term microbiota	7.944	1.623	0.316	4.896	0.000	4.747	11.140
Self‐assessment on microbiota knowledge	5.031	1.909	0.126	2.636	0.009	1.270	8.791
		***R*** ^***2***^ **= 0.527; *p* < 0.001**					

*Note:* Department: 0‐non nutrition and dietetics; 1‐nutrition and dietetics; Knowing the term of microbiota: 0‐no, 1‐yes; Evaluating the level of knowledge of the term microbiota: 0‐average and poor, 1‐good.

Abbreviation: SE, standard error.

## 4 Discussion

The microbiota includes all microorganisms found in humans, emphasizing its significant impact on health that cannot be disregarded (Çıtar Dazıroğlu et al. 2024). Awareness of the impact of microbes, particularly microbiota, on human health is crucial for individuals and communities. This insight can shape personal lifestyle decisions and assist policymakers in developing informed strategies (Abu‐Humaidan et al. 2021; Timmis et al. 2019). In this descriptive and analytical study, the effect of university students' attitudes towards healthy eating on their microbiota awareness and the relationship between microbiota awareness and several factors was examined. To the best of our knowledge, this is the first study to evaluate the relationship between microbiota awareness, healthy eating attitudes, and sociodemographic characteristics of Turkish university students. The primary outcomes of this study indicated that, compared to students from other departments, a more significant number of nutrition and dietetics students were familiar with microbiota, prebiotics, and probiotics, and their knowledge level was assessed as good. Nutrition and dietetics students had significantly higher ASHN and microbiota awareness total scores and subscale scores than students from other departments, while their BMI values were lower. Moreover, the current study determined that factors such as sex, department, receiving nutrition education, knowledge of microbiota, prebiotics, and probiotics, as well as a good knowledge of these terms, and ideally high healthy nutrition attitude were effective factors in awareness of microbiota.

Dietetics students tend to exhibit healthier eating attitudes than their peers in non‐nutrition‐related fields. This can be attributed to their specialized education, which focuses on eliminating unhealthy eating behaviors, encouraging healthy eating habits, optimizing food resource use, and enhancing skills in meal planning and weight management (Yolcuoğlu and Kızıltan 2022). During nutrition and dietetics training, students are taking compulsory courses, including general microbiology, food microbiology, microbiota and nutrition, assessment of nutritional status in the community, principles of nutrition, nutritional biochemistry, pathophysiology of chronic disease, and how to assess and modify dietary behaviors, attitudes, and patterns during their 4‐year education (Boak et al. 2022; Budhiwianto et al. 2023; Lawlis et al. 2019). The courses address food and nutrition to promote health, prevent and manage diseases, and optimize the health of individuals, groups, and populations. They also explore the human body's flora, pathogenic and non‐pathogenic microorganisms, and their interactions across various themes. Furthermore, in departments with non‐nutrition and dietetics programs, students are limited to nutrition courses, which are available for only half a semester and offered as elective options. In the current study, compared to students in other departments, the number of nutrition and dietetics students who heard about and knew the concepts of microbiota, prebiotics, and probiotics and assessed their self‐assessment of these topics as good was statistically significantly higher in this study.

Matusik et al. (Matusik et al. 2022) observed that dietetic students had significantly lower BMI values than non‐dietetic students. Yolcuoğlu and Kızıltan (Yolcuoğlu and Kızıltan 2022) found that the mean BMI values in females enrolled in the nutrition and dietetics department were lower compared to those in other fields of study. They also found that students in the nutrition and dietetics program were less likely to be underweight and obese in both genders based on their BMI categorization. Another study found that the gender and major of students in the faculty of health sciences significantly impacted their BMI (Özenoğlu et al. 2018). In our study, students in nutrition and dietetics exhibit a significantly lower BMI than their peers in other departments. Nutrition and dietetics students exhibit a higher prevalence of normal body weight compared to students in other departments, while the incidence of overweight and obese students is lower. However, the number of underweight students in nutrition and dietetics is higher. Research involving dietitians and undergraduate nutrition students revealed a prevalence of anti‐fat attitudes among them (Cassiano et al. 2021; Swift et al. 2013). In this context, it was thought that undergraduate dietetics students might have stigmatizing and prejudiced beliefs regarding individuals with higher body weights, with being thin potentially perceived as a marker of expertise.

Understanding attitudes towards healthy eating during young adulthood, when eating habits change and develop, is crucial for maintaining and improving health (Lee et al. 2022). Therefore, it is essential to assess university students' attitudes towards healthy eating. Research shows that nutrition and health education positively affect students' healthy eating attitudes (Seref et al. 2023; Özenoğlu et al. 2018; Topaktaş and Çetin 2023). A study including students from midwifery and nursing departments revealed a mean ASHN score of 75.5 ± 8.8, with no significant differences observed between the departments (Topaktaş and Çetin 2023). A study by Betül et al. involving health sciences students revealed that the mean ASHN total scores (70.6 ± 10.9) for nutrition and dietetics students were significantly higher than those of other departments (Seref et al. 2023). Iskender et al. found that students in health sciences who received nutrition education showed attitudes towards nutrition that were significantly higher than those who did not (Iskender et al. 2024). A study at 19 Mayis University in Türkiye revealed that students in the Department of Nutrition and Dietetics had higher total ASHN scores than students from other departments (Özenoğlu et al. 2018). In our study, the mean ASHN total score among students was 73.3 ± 10.36, with the highest mean sub‐dimension score recorded in positive nutrition at 21.7 ± 3.00. This study found that the total ASHN and subdimension scores of nutrition and dietetics students were significantly higher than those of students in other departments. The results of our study agree with the current literature. Depending on nutrition education, it is expected that students in the nutrition and dietetics departments would have higher attitude scores towards healthy eating. Health education was believed to influence students' attitudes towards healthy eating positively.

Previous research has shown that gender, education, knowledge level, and nutritional literacy are associated with awareness of microbiota (Bozkurt and Arslan 2023; Pehlivan 2020; Salari et al. 2020; Ülker et al. 2024). Studies among university students in Türkiye indicated that female microbiota awareness scores (Bozkurt and Arslan 2023) and knowledge about probiotics levels (Zemzemoğlu et al. 2019) were significantly higher than those of males. Nursing and midwifery students exhibited lower levels of microbiota awareness. They scored lower in various sub‐dimensions, including general knowledge, product knowledge, chronic illness, and knowledge of probiotics and prebiotics, compared to nutrition and dietetics students (Yılmaz 2023). People with medical degrees and university students who have completed a microbiology course have been shown to know more about microbiota and prebiotics (Abu‐Humaidan et al. 2021; Ayyash et al. 2021). In our study, microbiota awareness and subdimension scores were higher among female students studying nutrition and dietetics, receiving nutrition education, understanding microbiota, prebiotics, and probiotics, self‐assessment of these terms knowledge as good, and having an ideally high healthy eating attitude. These findings suggest that female gender, nutrition, and dietetics education positively influence microbiota awareness.

In the current study, it was determined that higher overall microbiota awareness scores among nutrition and dietetics students and all students were associated with higher attitudes towards healthy eating. Furthermore, the “Microbiota Awareness” total score and sub‐factor scores of the “Attitude Scale for Healthy Nutrition were found to be positively correlated with information on nutrition” and “positive nutrition,” while a negative correlation was observed with the malnutrition sub‐factor score. The information on nutrition sub‐factor relates to knowledge of food content and its health implications; the positive nutrition sub‐factor is associated with meal regularity and consistent intake of vegetables and fruits. Conversely, the malnutrition sub‐factor of ASHN is related to eating junk food and skipping main meals (Şahin‐Bodur et al. 2024). While no studies directly compare these parameters, our findings may indicate that participants with high healthy eating attitudes are more aware of microbiota.

Current literature indicates limited studies investigating university students' knowledge of prebiotics and probiotics and their awareness of the microbiota of BMI. However, research in the United States with 1497 people between the ages of 21 and 37 found that those who were normal weight were 35.4% more aware of and used probiotics than those who were underweight and 20.7% more than those who were overweight or obese (Kolady et al. 2018). A study carried out in Türkiye showed that overweight students exhibited a statistically significant lower level of awareness regarding microbiota compared to other students based on BMI (Hamurcu and İsmailoğlu 2022). Conversely, other studies indicated no differences in microbiota awareness levels when classified by BMI (Bozkurt and Arslan 2023; Davarci and Davarci 2025). Our study demonstrated that the overall microbiota awareness score and the subscale scores for students with normal body weight were higher than those of their overweight and obese colleagues; however, the differences were not statistically significant. Nevertheless, a notable negative correlation was observed between the microbiota awareness scores and BMI in overall university students. These results indicate that knowledge and awareness of the microbiota could contribute to maintaining and improving overall health and maintaining a healthy body weight. According to BMI classification, most university students within the normal BMI range may have been affected by the absence of significant differences in microbiota awareness levels.

Dietary interventions, including the intake of probiotics and prebiotics, can modulate gut microorganisms. Consumption of prebiotics and probiotic foods serves as a dietary approach to modify the gastrointestinal microbiota for health benefits (Tae and Kim 2024). A study conducted in Türkiye among health sciences students revealed that the nursing department consumed the most probiotic foods. Additionally, another study indicated that university students with greater knowledge of probiotics tended to consume more probiotic foods (Horasan et al. 2021; Zemzemoğlu et al. 2019). The study involving nutrition and dietetics students revealed a positive correlation between the students' knowledge levels and their consumption of probiotic foods, as well as their attitudes towards probiotic foods (Özgür and Dinçoğlu 2023). A study in Nigeria showed that medical science practitioners and students had a poor understanding of the advantages of probiotics and their use (Chukwu et al. 2015). In this study, the frequency of probiotic and prebiotic food consumption was higher among nutrition and dietetics students than in other departments. The outcomes may be influenced by education in nutrition and dietetics, students' knowledge levels, nutrition and health literacy, and income levels.

The gut microbiota influences the host's dietary responses, while the host can also alter the gut microbiota through altering dietary habits. The gut microbiota's composition, variety, and biodiversity can be influenced and modified by eating habits throughout life. Unhealthy eating patterns, particularly those associated with Western‐style diets, significantly contribute to gut microbial imbalance, chronic inflammation, and the prevalence of non‐communicable diseases, including obesity, diabetes, cancer, and cardiovascular disease (Dalamaga and Tsigalou 2024; Thomas et al. 2022). Research with university students across various countries indicates that the intake of vegetables and fruits among students is notably low, whereas their consumption of high‐fat foods, added sugars, and alcohol is significantly higher (Alves et al. 2020; Dolatkhah et al. 2019; Savage et al. 2024). Research indicates that university students tend to engage in unhealthy eating behaviors. Implementing healthy eating habits from adolescence to adulthood is important for decreasing the risk of chronic diseases in adulthood (Ushula et al. 2022). Thus, assessing the eating habits, microbiota awareness, and attitudes towards healthy nutrition among university students is crucial.

The study has the following strengths: it is the first study in which the relationship between microbiota awareness and healthy nutrition in Turkish university students is indicated, and the factors (age, gender, department of education, and BMI) that affected them are also clarified; and it is a study that determined that female gender, nutrition and dietetics education, normal body weight, and high/ideally high healthy nutrition attitude improves microbiota awareness. However, certain limitations exist within our study. First, self‐reporting was used to collect the data, which raises the possibility of recall bias and false reporting. Second, measurements of the participants' height and body weight were recorded based on the self‐reports. Third, the sample consists mainly of females. Fourth, the food frequency questionnaire form focused solely on the frequency of consumption, omitting any inquiries regarding the amounts of foods consumed. Implementing and performing more extensive observational studies will address this gap in the literature. Finally, this study sample included university students in four departments, so these results cannot be generalized to all students worldwide. As a result, it is important to repeat the research with students studying in different countries and departments.

## 5 Conclusions

In the present study, microbiota awareness was higher among nutrition and dietetics students; nutrition education and a highly healthy nutrition attitude positively affect microbiota awareness. In addition, nutrition and dietetics students had significantly higher healthy nutrition attitudes and microbiota awareness than students from other departments, while their BMI values were lower. Moreover, the current study determined that factors such as female gender, knowledge of microbiota, prebiotic, and probiotic, and a good knowledge of these terms and ideally high healthy nutrition attitude were effective factors in awareness of microbiota. Another significant study result was that some probiotic and prebiotic foods consumption was higher among undergrad dietetics students. Interventions aimed at increasing nutritional knowledge and promoting healthy eating habits in the university population may have potential benefits on microbiota awareness and attitude towards healthy nutrition. Given the association between microbiota and several diseases and health conditions, it highlights the necessity of dissemination of this knowledge. Therefore, it is recommended that the topic of healthy nutrition and microbiota be incorporated into the educational curricula of all university departments while enhancing knowledge through scientific conferences and academic research.

## Author Contributions

**Emine Kocyigit:** conceptualization (equal), data curation (equal), formal analysis (lead), investigation (lead), writing – original draft (lead), writing – review and editing (lead). **Ayçıl Özturan Şirin:** data curation (equal), visualization (equal), writing – original draft (equal), writing – review and editing (equal). **Nilüfer Ozkan:** conceptualization (equal), data curation (supporting), writing – original draft (supporting), writing – review and editing (supporting).

## Ethics Statement

Ethical permission was obtained from the Ordu University Social Sciences and Humanities Research Ethics Committee (Meeting Number: 10; Decision Number: 2023‐245; Date: 28.11.2023). The research was conducted in compliance with the principles outlined in the Declaration of Helsinki. All participants provided informed consent.

## Conflicts of Interest

The authors declare no conflicts of interest.

## Data Availability

The datasets generated and analyzed during the current study are available from the corresponding author on a reasonable request.
